# The emerging role of the gut microbiota in vaccination responses

**DOI:** 10.1080/19490976.2025.2549585

**Published:** 2025-08-30

**Authors:** V. Decker, K. Qureshi, L. Roberts, N. Powell, JR. Marchesi, BH. Mullish, JL. Alexander

**Affiliations:** a Division of Digestive Diseases, Department of Metabolism- Digestion and Reproduction, Imperial College London, London, UK; b Gastroenterology and Hepatology Imperial College NHS Healthcare Trust, London, UK; c St Mark’s Hospital The National Bowel Hospital and Academic Institute, IBD Unit, London, UK

**Keywords:** Gut microbiome, immunity, parenteral vaccines, COVID-19, vaccine efficacy

## Abstract

The gut microbiota has emerged as a key modulator of host immune responses, and growing evidence suggests it plays a role in shaping vaccine-induced immunity. While immunization remains vital for preventing infectious diseases, inter-individual variability in vaccine responses poses a persistent challenge. Traditional factors such as age, sex, genetics, and immune status do not fully account for this variability. Recent studies highlight the gut microbiome as a potential contributor. This review examines current evidence linking the gut microbiota to vaccine responses, with a focus on vaccines against SARS-CoV-2, hepatitis B virus, and influenza. Human studies show associations between microbial composition, particularly taxa like *Bifidobacterium adolescentis*, and immunogenicity. Microbial metabolites, such as short-chain fatty acids and bile acids, influence T-cell differentiation, antibody production, and cytokine responses. Factors that alter microbiota composition, including antibiotics, diet, and prebiotic or probiotic supplementation, can impact vaccine responses, highlighting a dynamic gut-immune relationship. Experimental models further support these observations, showing diminished responses in germ-free or antibiotic-treated animals and enhanced responses following microbial-based interventions. These findings also suggest the gut microbiota may be harnessed to improve vaccine efficacy. Future research should explore the potential for microbiota-targeted strategies to optimize vaccine efficacy, particularly in immunocompromised populations.

## 1. Introduction

Vaccinations are essential in the prevention and eradication of many infectious diseases. Their efficacy is exemplified by the case of measles, where there were more than 460,000 cases and 90 deaths in the UK per year before vaccination was introduced.^1^ In 2023, there were 216 confirmed cases and no deaths. It is believed that since vaccines were made available globally by the World Health Organization in 1974, 154 million deaths have been prevented.^2^

Both humoral and cellular immune responses are necessary for vaccine-induced immunity.^3^ Vaccination stimulates the generation of cross-reactive T-cells, such as CD4^+^ and CD8^+^, which enable the immune response to infections such as SARS-CoV-2, mitigating COVID-19 severity.^4,5^ T-helper (Th) 17 cells, a subset of CD4^+^ cells, are particularly reactive against pathogens that enter the body through mucosal tissue, including most respiratory and gastrointestinal infections.^6^ They recruit neutrophils and drive Th1 immunity by releasing interleukin (IL) 17 and antimicrobial peptides. T-regulatory (Treg) cells, which are more abundant in older age, are thought to diminish the immune response to vaccinations, contributing to poorer vaccine-induced immunity in the elderly.^7^ B-cells produce antibodies and form memory cells, which allow the production of a rapid immune response when re-encountering an antigen.^8^ The antibodies produced prevent the spread of intracellular infections and cause rapid destruction of extracellular microorganisms.

Vaccinations induce varying levels of immune response, and post-vaccination breakthrough infection rates and severity of infection vary greatly between seemingly well-matched individuals.^9,10^ It is well known that immune responses to vaccinations, commonly measured using immunoglobulin (Ig) G1 levels, may vary depending on a multitude of intrinsic and extrinsic factors. Age is a key determinant of response, which is demonstrated by higher IgG levels in infants receiving the measles vaccination at the age of six months versus nine months.^11,12^ In general, the younger healthy adult population has higher antibody responses than the elderly, which can be observed with the influenza vaccine.^13^ Another well-reported factor is sex: Females have higher antibody responses to certain vaccinations, such as Hepatitis B.^14^ Other known factors for certain vaccines are genetics, such as blood group antigens, immunosuppressive therapies, like long-term steroid use or biologics in immune-mediated inflammatory disorders, and comorbidities, as with children with celiac disease.^15,16^ However, these factors do not fully account for the variability in immune response.

During infancy, the colonization of the gut by microbes is known to affect the maturation of the early immune system.^17^ In adults, microbiota composition impacts adaptive and innate immune homeostasis.^18^ Research has also shown that drug therapies can be affected by microbiota composition. Giving a fecal microbiota transplant (FMT) to cancer patients prior to immunotherapy is associated with a better overall prognosis.^19^ Moreover, the gut microbiota produces a multitude of metabolites with immuno-modulatory functions, such as short-chain fatty acids (SCFAs), branched-chain fatty acids (BCFAs) and secondary/tertiary bile acids.^20^ SCFAs influence epithelial barrier integrity, immune cell metabolism and differentiation and cytokine production (IL-10).^21,22^ Bacterially derived bile acids can directly influence host immunity, as seen in the downregulation of Th17 differentiation by 3-oxolithocholic acid and isolithocholic acid.^23^ As we will discuss in this review, emerging data from animal and human studies suggest that the gut microbiome may be an important factor influencing immune responses to vaccination, especially through the production of immunomodulatory metabolites. Whilst there is an existing body of literature reviewing associations between the gut microbiome and responses to orally delivered vaccines,^24^ the gut microbiota also exerts its influence on immune function distant to the gut mucosal interface. Prior reviews have outlined the emerging relationship between the human microbiome and vaccine immunogenicity, focusing particularly on early life and global health settings.^25^ Additionally, others have been published focusing on COVID-19 vaccination.^26,27^ In the current review, we build upon this foundation and further examine and synthesize both mechanistic and clinical data linking the gut microbiome to immune responses to multiple parenterally delivered vaccines across diverse population groups and animal models.

## 2. Human studies

### 2.1. Overview

A number of human studies have been performed in recent years assessing the composition and function of the gut microbiota in the context of parenterally delivered vaccination. These include sampling of patients receiving COVID-19 vaccines, Bacillus Calmette-Guérin (BCG), Tetanus Toxoid (TT), Hepatitis B (HBV), Oral Polio Vaccine (OPV), PCV13 (pneumococcal), Infanrix Hexa (Diphtheria, Tetanus, Pertussis, Polio, Hepatitis B, Haemophilus influenzae type b), Meningococcal B, Pneumococcal capsular polysaccharide (PCP) and influenza vaccines (Table 1).

**Table 1.** Summary of key observational human studies on associations of gut microbiota with parenteral vaccine immune responses.

Study	Date	Vaccine(s) name	Vaccine(s) type	Population	Healthy or diseased population	Location	Methods for microbiota analysis	Methods for immunogenicity analysis	Associated microbiota (taxa associations)
Ng *et al*.^28^	2022	Sinovac and BNT162b2	Sinovac: Inactivated virus vaccine BNT162b2: mRNA vaccine	37 adults (Sinovac) 101 adults (BNT162b2)	Healthy	Hong Kong	Stool shotgun metagenomic sequencing	SARS-CoV-2 surrogate virus neutralization test (sVNT) Anti-SARS-CoV-2 Spike-receptor binding domain (RBD) IgG ELISA	Sinovac: ↑ *Bifidobacterium adolescentis* associated with ↑ neutralizing antibodies ↑ *Bacteroides vulgatus, Bacteroides thetaiotaomicron* & *Ruminococcus gnavus* associated with ↓ neutralizing antibodies
									BNT162b2: ↑ *Bifidobacterium adolescentis* associated with ↑ neutralizing antibodies ↑ *Eubacterium rectale, Roseburia faecis* & two Bacteroides species (*Bacteroides thetaiotaomicron* & *Bacteroides* sp OM05–12) associated with ↑ neutralizing antibodies
Jia *et al*.^29^	2022	BBIBP-CorV vaccine	Inactivated virus vaccine	95 adults	Healthy	China	16S rRNA gene sequencing	SARS-CoV-2 sVNT Anti-SARS-CoV-2 Spike-receptor binding domain (RBD) IgG ELISA	↑ Bacteroides & Parabacteroides associated with ↑ IgG
Han *et al*.^30^	2022	Sinovac	Inactivated virus vaccine	30 adults	Healthy	China	Stool shotgun metagenomic sequencing	SARS-CoV-2 sVNT Anti-SARS-CoV-2 Spike-receptor binding domain (RBD) IgG ELISA	↑ *Clostridium leptum, Lactobacillus ruminis* & *Ruminococcus toques* correlated with ↑ IgG/IgM ↑ *Segatella copri* correlated with ↓ IgG
Tang *et al*.^31^	2022	BBIBP-CorV vaccine	Inactivated virus vaccine	207 adults	Healthy	China	Stool shotgun metagenomic sequencing	Virus-specific IgG/IgM Chemiluminescent Immunoassay (CLIA) ACE2-RBD inhibiting antibody CLIA	↑ *Collinsella aerofaciens* & *Veillonella dispar* associated with ↑ ACE2-RBD inhibiting antibodies ↑ *Lawsonibacter asaccharolyticus* correlated with ↓ ACE2-RBD inhibiting antibodies
Alexander *et al*. ^32^	2023	ChAdOx1 nCoV-19 or BNT162b2 vaccine	ChAdOx1 nCoV-19: Viral vector vaccine BNT162b2: mRNA vaccine	15 adults (ChAdOx1 nCoV-19) 28 adults (BNT162b2)	Inflammatory bowel disease (IBD) patients	UK	16S rRNA gene sequencing Metabolomic profiling using 1 H NMR Bile acid profiling using UHPLC-MS	Anti-SARS-CoV-2 Spike-receptor binding domain (RBD) antibody CLIA	↑ *Bilophila* associated with ↑ antibody response ↑ Streptococcus associated with ↓ antibody response.
Hirota *et al*.^33^	2023	BNT162b2	BNT162b2: mRNA vaccine	95 adults	Healthy	Japan	16S rRNA gene sequencing	Anti-SARS-CoV-2 Spike IgG ELISA	No significant correlations between these taxa and vaccine-induced antibody or T cell responses after adjustments for age, sex, and stool sampling timing.
Daddi *et al*.^34^	2023	BNT162b2 and SpikeVax	BNT162b2: mRNA vaccine Spikevax: mRNA vaccine	9 adults (BNT162b2) 7 adults (Spikevax)	Healthy	USA	16S rRNA gene Sequencing	Anti-SARS-CoV-2 Spike IgG. ELISA	↑ *Bilophila (*Desulfobacterota) correlated with ↑ IgG ↑ *Colidextribacter, Clostridium innocuum, Lachnoclostridium, Bacteroides* & an unclassified *Lachnospiraceae* genus with ↓ IgG
Peng *et al*.^35^	2023	BNT162b2 and Sinovac	BNT162b2: mRNA vaccine Sinovac: Inactivated virus vaccine	121 adults (BNT162b2) 40 adults (Sinovac)	Healthy	Hong Kong	Stool shotgun metagenomic sequencing	SARS-CoV-2 sVNT	BNT162b2: ↑ *Bifidobacterium adolescentis, Bifidobacterium bifidum*, & *Roseburia faecis* associated with ↑ neutralizing antibodies
									Sinovac: ↑ *Phocaeicola dorei, Blautia massiliensis*, & *Dorea formicigenerans* associated with ↑ neutralizing antibodies ↑ *Faecalibacterium prausnitzii* associated with ↓ neutralizing antibodies
Lunken *et al*.^36^	2023	BNT162b2	BNT162b2: mRNA vaccine	52 adults	Healthy	Canada	16S rRNA gene Sequencing	Anti-SARS-CoV-2 Spike IgG CLIA ACE2-RBD inhibiting assay (CLIA)	↑ *Bifidobacterium animalis, Bacteroides plebeius* & *Bacteroides ovatus* associated with ↑ IgG avidity ↑ *Bifidobacterium bifidum* & *Akkermansia intestini* associated with ↓ IgG avidity ↑ *Prevotella, Haemophilus, Veillonella* & *Ruminococcus gnavus* associated with ↑ antibody production
Ray *et al*.^37^	2023	BNT162b2	BNT162b2: mRNA vaccine	180 adults	90 healthy 90 people living with HIV (PLWH)	Sweden	16S rRNA gene Sequencing	Anti-SARS-CoV-2 Spike IgG CLIA CD4+ T cell assay Flow cytometry (CD4+ and CD8+ T-cell counts and HIV viral load)	↑ *Bifidobacterium and Faecalibacterium* associated with ↑ IgG ↑ *Agathobacter, Lactobacillus, Bacteroides*, & *Lachnospira* correlated with ↑ IgG and CD4+ T cells ↑ *Methanobrevibacter*, Ruminococcaceae DTU089, *Marvinbryantia, Cloacibacillus*, & *Succinivibrio* associated with ↓ IgG
Seong *et al*.^38^	2024	BNT162b2 and ChAdOx1	BNT162b2: mRNA vaccine ChAdOx1 nCoV-19: Viral vector vaccine	23 adults (BNT162b2)* 21 adults (ChAdOx1)* *all 44 received BNT162b2 booster	Healthy	South Korea	16S rRNA gene and shotgun metagenomic sequencing	Anti-SARS-CoV-2 Spike IgG CLIA	BNT162b2: ↑ *Faecalibacterium prausnitzii* associated with ↑ IgG ↑ *Faecalibacterium prausnitzii Prevotella_uc*, & PAC001304_s *(Prevotella), G. formicilis, Bacteroides dorei*, and *Anaerostipes rectalis* associated with ↑ antibody half-life ChAdOx1: ↑ *Escherichia coli, Alistipes, Parabacteroides* & *Enterococcus* associated with ↑ antibody half-life
Ng *et al*.^39^	2025	BNT162b2	BNT162b2: mRNA vaccine	242 adults	Healthy	Hong Kong	Shotgun metagenomic sequencing	Chemiluminescent microparticle immunoassay	↑ *Ruminococcus bicirculans, Phocaeicola excrementihominis and Streptococcus salivarius* associated with ↑ neutralizing antibodies ↑ *Bacteroides thetaiotaomicron* associated with ↓ neutralizing antibodies
Huda *et al*.^40^	2014	BCG, TT, HBV, Oral Polio Vaccine (OPV)	BCG: Live attenuated vaccine TT: Toxoid vaccine HBV: Recombinant subunit vaccine OPV: Live attenuated vaccine	48 infants	Healthy	Bangladesh	16S rRNA gene sequencing	Polio-specific IgA and IgG, HBV-specific IgG and TT-specific IgG Enzyme-Linked Immunosorbent Assay (ELISA) Flow cytometric assay of specific cell-mediated immune response in activated whole blood (FASCIA)	↑ Actinomycetota associated with ↑ T-cell responses to BCG, OPV, and TT & TT- and Purified Protein Derivative (PPD)- specific IgG ↑ *Enterobacteriales* associated with ↓ T-cell responses to TT & PPD-specific IgG *Pseudomonadales* associated with ↓ T-cell responses to HBV, OPV, and TT *Clostridiales* associated with ↓ T-cell responses to TT
Huda *et al*.^41^	2019	BCG, TT HBV, Oral Polio Vaccine (OPV)	BCG: Live attenuated vaccine TT: Toxoid vaccine HBV: Recombinant subunit vaccine OPV: Live attenuated vaccine	291 infants	Healthy	Bangladesh	16S rRNA gene sequencing method	Polio-specific IgA and IgG, HBV-specific IgG and TT-specific IgG ELISA FASCIA	BCG: ↑ *Bifidobacterium* in early infancy associated with ↑ CD4+ response at 2 years TT: ↑ *Bifidobacterium* in early infancy associated with ↑ CD4+ response at 15 wks, and ↑ IgG response at 2 yrs HBV: ↑ *Bifidobacterium* in early infancy associated with ↑ CD4+ response at 15 wks, and ↑ CD4+ response and IgG response at 2 yrs OPV: ↑ *Bifidobacterium* in early infancy associated with ↑ IgG response at 15 wks, and ↑ IgA and IgG response at 2 yrs
Moroishi *et al*.^42^	2022	Pneumococcal capsular polysaccharide (PCP) and TT vaccines	PCP: polysaccharide vaccine TT: Toxoid vaccine	133 infants (TT) 22 infants (PCP)	Healthy	USA	16S rRNA gene sequencing	Anti-PCP Ig assay TT IgG ELISA	TT: ↑ *Aeriscardovia aeriphila* associated with ↓ TT IgG
Ryan *et al*.^43^	2025	PCV13, Infanrix Hexa (Diphtheria, Tetanus, Pertussis, Polio, Hepatitis B, *Haemophilus influenzae* type b) and Meningococcal B	PCV13: conjugate vaccine Diphtheria: Toxoid vaccine TT: Toxoid vaccine Pertussis: Acellular subunit vaccine Polio: Inactivated vaccine Hepatitis B: Recombinant subunit vaccine *Haemophilus influenzae* type b: Conjugate vaccine Meningococcal B: Recombinant subunit vaccine	191 infants	Healthy	Australia	Shotgun metagenomic sequencing	Vaccine-specific IgG titers (ELISA) Opsonophagocytic assay (functional antibody response) Serum bactericidal assay	↓ *Bifidobacterium* species (*Bifidobacterium breve, Bifidobacterium pseudocatenulatum* and *B. bifidum*) at week 6 (neonatal antibiotic group) correlated with ↓ IgG titers against PCV13 polysaccharides (esp. PPS6B) and *Haemophilus influenzae* type b and diphtheria toxoid antigens

These studies have generally analyzed baseline microbiota using stool samples collected prior to vaccination and vaccine immunogenicity using blood samples collected after immunization to determine associations between microbiota composition and immune responses.

**Figure 1.**
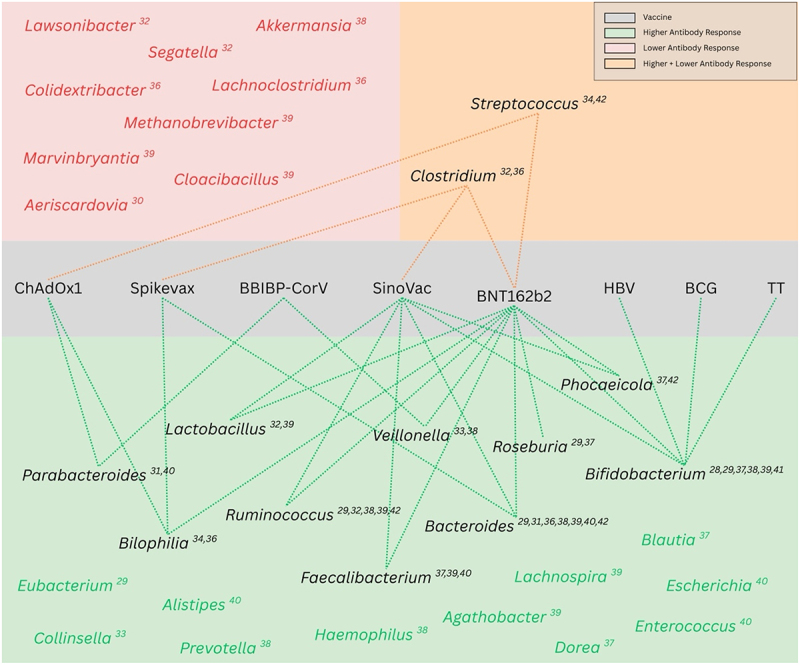
Diagram representing microbiota-vaccine associations and antibody responses This diagram shows reported links between specific gut microbiota genera and immunogenic response for various vaccines. Genera associated with lower antibody responses are pictured in the red section, genera associated with both lower and higher antibody responses are pictured in the orange section and genera associated with higher antibody response are pictured in the green section, with the vaccines implicated in the gray section. Microbes supported by only single studies are included but displayed without connections to reduce visual complexity.

These studies consistently report significant associations between gut microbiota and parenteral vaccine immunogenicity, and some associations appear to be common across studies and vaccine types (Figure 1).

### 2.2. COVID-19 vaccines

Several studies have explored associations between the pre-vaccination gut microbiota and responses to COVID-19 vaccines. Ng *et al*. investigated the impact of microbiota composition on the immune response to Sinovac and BNT162b2, stratifying participants into high and low responders according to antibody titers.^28^ High responders of the Sinovac vaccine showed a high baseline abundance of *Bifidobacterium adolescentis* relative to low responders, and a reduction of *Bacteroides vulgatus* one month post vaccination, with the latter correlating with antibody production. Similarly, within the baseline microbiota of the BNT162b2 cohort, *B. adolescentis* was enriched in the high responders, alongside *Eubacterium rectale*, *Roseburia faecis* and two *Bacteroides* species (*Bacteroides thetaiotaomicron* and *Bacteroides sp OM05–12)*. In the BBIBP-CorV vaccine, high levels of *Bacteroides* and *Parabacteroides* in the commensal gut microbiota of human participants were also associated with increased IgG production.^29^ Meanwhile, Tang *et al*. found high responders to the BBIBP-CorV vaccine exhibited a high abundance of baseline *Collinsella aerofaciens* and *Veillonella dispar* relative to low responders, both of which correlated with increased IgG production.^31^ Another study examined microbiota changes to the BNT162b2 vaccine in healthy controls and people living with HIV (PLWH).^37^ They found increased levels of *Bifidobacterium* pre-vaccination correlated with higher IgG titers, an association that held true in the PLWH cohort even in the context of HIV and antiretroviral therapy-related gut dysbiosis.^44–46^

Following Ng *et al*.’s work, the same team conducted a subsequent analysis to investigate whether baseline microbiota could predict longer-term immunity, at six months post vaccination.^35^ In the BNT162b2 group, high responders at six months showed a consistent abundance of *B. adolescentis* alongside *B. bifidum* and *R. faecis*. The Sinovac cohort showed a different pattern, with high responders characterized by increased *Phocaeicola (Phocaeicola dorei)*, and lower levels of *Faecalibacterium prausnitzii*. In contrast, *F. prausnitzii* was the most abundant species significantly associated with prolonged antibody half-life in BNT162b2 vaccinees.^38^ These findings suggest that different vaccine types, with their distinct antigen presentations and adjuvant systems, may activate different immune pathways that are mediated by specific bacterial taxa. A secondary outcome measure in the Ng *et al*. study explored the correlation between microbiota and vaccination-related adverse effects, namely injection site pain, fatigue, fever, myalgia, drowsiness, headache and chills. Notably, two *Megamonas* species and *Segatella copri* correlated with reduced adverse effects following Sinovac and BNT162b2 vaccines.^28^ Interestingly, a separate cohort study of BNT162b2 vaccinees in Canada found higher levels of *Prevotella* pre-vaccination were associated with higher IgG levels.^36^ In fact, the taxa found to be more abundant in high responders in the Canadian cohort differed significantly from those in Ng *et al*.’s Chinese cohort, including *R. gnavus*, which Ng *et al*. had found to be associated with lower antibody response in their 2022 study. Ng *et al*.’s follow up study identified *Ruminococcus bicirculans* as particularly abundant in BNT162b2 high responders.^39^ Han *et al*. found a different *Ruminococcus species* (*R. toques*) in higher abundance in high responders to the Sinovac vaccine, alongside *Clostridium leptum and Lactobacillus ruminis*.^30^ However, within this study, *S. copri* correlated with decreased IgG production.

An intriguing pattern emerges regarding the role of pro-inflammatory bacteria in vaccine immunogenicity. *Bilophila*, typically associated with IBD,^47^ consistently correlated with enhanced antibody responses across multiple studies. In a UK study of 42 anti-TNF-treated patients with IBD receiving either BNT162b2 or ChAdOx1 vaccines, *Bilophilia* levels at baseline were correlated with final IgG titers.^32^ A similar finding was reported in a US-based study, where elevated *Bilophila (Desulfobacterota*) levels in cohorts receiving BNT162b2 and Spikevax vaccines were correlated with higher IgG titers.^34^ Collectively, these studies highlight that a microbiota with pro-inflammatory genera, such as *Bilophila*, may prime immune responses and enhance vaccine immunogenicity, particularly in populations with underlying inflammatory conditions. In contrast, *S. copri* displays a more complex immunological profile. Whilst implicated in numerous disease processes, including inflammatory bowel disease,^48^ non-small cell lung cancer,^49^ rheumatoid arthritis^50^ and inflammation secondary to HIV,^51^ in some Crohn’s disease pre-clinical models, *S. copri* was found to be less abundant than in healthy controls.^52^ In colorectal cancer patients, significant depletion of *S. copri* was associated with an aberrant microbiota profile,^53^ highlighting its potentially pleomorphic role in microbial-immune interplay. Indeed, in the context of vaccine response, *S. copri* was associated with reduced adverse effects but also correlated with lower antibody titers,^30^ indicating a potential trade-off between vaccine tolerability and immunogenicity.

Whilst most studies have focused on the association between baseline microbiota and vaccine response, other studies have looked at changes in the microbiota following vaccination. There are different patterns seen in gut microbiota changes post vaccination. A study investigating the BBIBP-CorV vaccine found that gut microbiota diversity decreased in healthy adults post vaccination.^31^ Similarly, Peng *et al*. found that in both BNT162b2 and Sinovac cohorts, participants had decreased abundances of Bacillota and Actinomycetota, and increased abundances of Bacteroidota and Pseudomonadota at 6 months. These results corresponded to a reduced Gram-positive to Gram-negative ratio of gut microbiota post-vaccination, which is a potential marker of an increased inflammatory gut status.^54^

In summary, studies suggest that specific gut bacteria may influence immune response to COVID-19 vaccines. High responders to Sinovac and BNT162b2 vaccines often showed elevated levels of *B. adolescentis* and *B. bifidum* among other SCFA-producers whilst *S. copri* correlated with fewer side effects but also lower antibody levels. Pro-inflammatory bacteria, such as *Bilophila*, were associated with enhanced antibody responses, especially in inflammatory conditions.

### 2.3. Other parenteral vaccines

Whilst the majority of studies on the microbiota and response to parenteral vaccines have been conducted in the context of the COVID-19 pandemic, several studies have addressed other vaccines, namely the BCG, TT, Hep B, influenza, PCV13 and Infanrix Hexa vaccines. As highlighted in the COVID-19 vaccine studies, *Bifidobacterium* is emerging as a potential modulator of vaccine immunogenicity, as many studies have demonstrated a positive correlation between *Bifidobacteria* and enhanced antibody production.^28,35–37^ Similarly, Huda *et al*. found the abundance of Actinomycetota, especially *Bifidobacterium*, was positively associated with BCG, TT and Hep B T cell responses.^40^ A later study by the same group corroborated these findings, with increased levels of *Bifidobacterium* correlating with increased IgG and CD4^+^ titers across the BCG, TT and Hep B vaccines, suggesting this bacterial genus may have a role in enhancing vaccine response across diverse antigens.^41^ Conversely, Moroishi *et al*. showed that an abundance of *Aeriscardovia aeriphila* was associated with reduced TT-specific IgG antibodies.

Studies in animal models have established a link between the gut microbiota and antibody responses to the influenza vaccine,^55^ which is now the subject of further investigation in humans. Hagan *et al*.’s seminal trial demonstrated the impact of antibiotics to modulate vaccine response by administering a five-day course of an oral broad-spectrum antibiotic regimen (neomycin, vancomycin, and metronidazole) to participants with no influenza exposure in the previous three years, before they received the influenza vaccine.^56^ Their immune responses were compared to controls. The antibiotic intervention hindered the antibody production against the H1N1 A/California-specific virus. The antibiotics disrupted secondary bile acid production, particularly of lithocholic acid (LCA), thereby amplifying inflammatory signaling pathways (e.g., AP-1) and impairing antibody response. Similarly, in a COVID-19 vaccine study, the use of pre-immunization antibiotic treatment was associated with a significant 74% lower rate of seroconversion of neutralizing antibodies after one dose of the BNT162b2 vaccine.^57^ These findings suggest that the immune-modulating properties of gut bacteria may not be universal, but rather context-dependent, influenced by factors such as vaccine type, microbial diversity and host health status.

A recent prospective study by Ryan *et al.*, following 191 infants from birth through 15 months, provided critical insights into the impact of antibiotic exposure timing on vaccine immunogenicity.^43^ Specifically, reduced abundances of *Bifidobacterium* species (*B. breve*, *B. pseudocatenulatum* and *B. bifidum*) at six weeks strongly correlated with lower antibody responses against polysaccharide antigens in PCV13 and Infanrix Hexa vaccine antigens at seven months. Functional analyses further confirmed impaired opsonophagocytic activity against PCV13 serotype PPS6B. Notably, these effects were specific to neonatal antibiotic exposure; intrapartum antibiotics did not significantly impact microbiota or vaccine responses. Ryan *et al*.’s findings extend prior observations made by Hagan *et al*., by explicitly linking antibiotic-induced microbiota disruptions to impaired vaccine responses and further highlighting the importance of the timing of microbiota disruption in determining subsequent vaccine efficacy. This study provides compelling new evidence that early-life reductions in *Bifidobacterium* significantly impact vaccine immunogenicity, reinforcing the central role of *Bifidobacterium* as a largely consistent predictor of enhanced vaccine response across various vaccines and age groups.

#### 2.3.1 Factors affecting vaccine efficacy

As this review collates a diverse group of human studies, it is essential to acknowledge the intrinsic and extrinsic factors, such as geographical differences, that can modulate human gut microbiota composition. A study by Subramanian *et al*. from 2014 showed that malnourished Bangladeshi children had immature microbiota, predominantly consisting of *Streptococcaceae*, *Fusobacteriaceae* and *Enterobacteriaceae*, whilst having reduced levels of *Lactobacillus* and *Bifidobacterium* compared to healthy age-matched children.^58^ The composition of the gut microbiota is highly variable between individuals, with notable distinctions between Westernized and non-Westernized populations. In rural African populations, the gut microbiota has higher concentrations of *Prevotella*, *Treponema*, and *Clostridia*, which is likely due to the individuals’ high fiber and plant-based diets, whilst European populations tend to have higher levels of *Bacteroidota* and lower overall microbiota diversity.^59^ Rothschild *et al*. found in 2018 that lifestyle and geography alone accounted for microbiota variation more than host genetics in more than 1000 individuals.^60^ Among our studies, Asian populations consistently exhibit beneficial associations between *B. adolescentis* and enhanced vaccine immunogenicity across multiple vaccine platforms,^28,35,41^ while Western populations show more heterogeneous *Bifidobacterium* relationships.^36,37,43^ Notably, certain taxa exhibit population-specific directional effects: *F. prausnitzii* is associated with enhanced responses in Swedish and South Korean cohorts^37,38^ but reduced response in the Hong Kong population.^35^ Population-level differences in microbiota are likely to impact immune system maturation and mucosal tolerance, which partially influence the host’s ability to mount robust vaccine responses.

Age is a key determinant of vaccine response, and the gut microbiota evolves dramatically across the lifespan: early life is characterized by instability and low diversity with a small number of dominant bacterial families, transitioning to adult-like composition by ages two to three.^61^ Adult microbiota exhibits high diversity and inter-individual heterogeneity with increased stability, while aging brings progressive decline in both diversity and stability.^62^ Among human studies, infant populations universally demonstrate positive *Bifidobacterium*-vaccine associations across geographic locations, while adult populations exhibit more variable and contradictory patterns. This disparity may suggest that the early-life microbiota’s instability and limited diversity align with vaccine efficacy through more predictable immune modulation, whereas the increased complexity of adult microbiomes manifests with more variable vaccine responses. Importantly, existing COVID-19 vaccine studies have focused exclusively on adults while other parenteral vaccine trials (BCG, TT, HBV, OPV, PCV13 and Infanrix Hexa) were conducted in infants, highlighting a fundamental limitation in extrapolating findings across life stages given that infant gut microbiota undergoes significant compositional and diversity changes during early development that may not translate to established adult microbiomes.^61^

Nonetheless, collectively, human studies highlight that *Bifidobacterium* demonstrates remarkable consistency as being positively associated with immune responses to vaccination across diverse vaccine platforms. High *Bifidobacterium* abundance (particularly *B. adolescentis*), presence of butyrate-producing taxa (*Faecalibacterium*, *Roseburia*), and context-dependent enrichment of pro-inflammatory genera (*Bilophila*) all show promise as modulators of vaccine-responsiveness. These exciting discoveries do not yet amount to a core microbiota signature of vaccine responsiveness, and further studies are needed accounting for geographical, age and disease-specific variation before such findings can be translated into clinical practice.

### 2.4. Microbiota-derived metabolites and metabolic pathways

In addition to evaluating microbiota composition, some studies have also explored the metabolites and metabolic pathways that may influence immune responses to vaccines.

Bacteria such as *E. rectale* and *R. faecis* are both SCFA producers and have been linked to an increased antibody response in BNT162b2 vaccinees,^28^ suggesting certain microbiota-derived metabolites may act as adjuvants to vaccines. Indeed, in the BBIBP-CorV cohort study, Tang *et al*. found that serum levels of SCFAs, namely acetic acid, butyric acid and isovaleric acid, correlated with increased antibody response at day 42 post vaccination.^31^ Notably, they found fecal and serum levels of SCFAs were higher in high responders compared to low responders, indicating they may have a role in improving antibody response post BBIBP-CorV vaccination.

In a BNT162b2 study, the group with a longer antibody half-life showed significant enrichment of *F. prausnitzii*, which is a key butyrate-producing bacterium.^38^ Other SCFA-producing bacteria, including *Prevotella* and *Bacteroides, Ruminococcaceae* and *Lachnospiraceae* were also notably abundant in this group. Similarly, Alexander *et al*. found that butyrate-producing bacteria (e.g., *Butyricicoccus*) and the BCFA, isobutyrate, were enriched in high responders.^32^ *Ruminococcaceae* emerges as a key player in vaccine response based on Ng et al.‘s recent findings of abundant *R. bicirculans* in BNT162b2 high responders.^39^ This genus synthesizes SCFAs including butyrate, propionate and acetate, with butyrate playing a specific role in boosting antibody production by upregulating follicular helper T cells necessary for plasma cell activation and differentiation.^63,64^ However, some bacteria with SCFA-producing potential were also abundant in low responders. Peng *et al*. found that butyrate, isobutyrate, isovalerate, and benzenebutanoic acid were elevated in low responders to the BNT162b2 vaccine.^35^ Moreover, high responders showed a different pattern of enriched metabolites, including nicotinic acid, γ-aminobutyric acid, fumaric acid, 2-aminoisobutyric acid, m-coumaric acid and threonic acid. In a subsequent BNT162b2 study, bacteria like *Megasphaera* spp., which are also prominent isovaleric and isobutyric acid producers, were associated with a poorer vaccine response.^36^ These seemingly contradictory findings may be explained by several factors: measurement timing, local versus systemic concentrations of metabolites, the specific bacterial species producing them, and overall microbiota composition context. The differential metabolite profiles between high and low responders suggest that immunomodulatory effects may depend on the broader metabolic milieu rather than individual SCFA concentrations alone. Supporting this notion, Lunken *et al*. found that high dietary fiber intake enhanced IgG binding strength maturation between BNT162b2 doses while reducing total BCFAs post-vaccination, suggesting dietary modulation of the microbiome influences vaccine response.^36^ These findings indicate that SCFA effects on COVID-19 vaccine immunogenicity are context-dependent, with no clear consensus on which specific fatty acids are consistently associated with antibody response.

When broad-spectrum antibiotic therapy was given to influenza vaccine recipients, changes in metabolic responses were observed, notably elevation of primary bile acids and a significant reduction in bacterially produced secondary bile acids, such as LCA, in comparison to non-antibiotic-treated controls.^56^ The authors postulate that this reduction in secondary bile acids promotes a pro-inflammatory response, akin to that seen in elderly adults receiving influenza vaccination, which is detrimental to vaccine immunogenicity. Recent research by Ryan *et al*. has further detailed inflammatory consequences following neonatal antibiotic exposure, and their potential link to reduced antibody response.^43^ Neonatal antibiotic exposure was associated with elevated baseline activity of inflammation-related blood transcriptional modules (BTMs). These BTMs, particularly monocyte-related, showed a negative correlation with several vaccine antibody responses measured at seven months post-vaccination. Furthermore, causal mediation analyses revealed that increased abundance of beneficial bacteria such as *B. breve* and *B. bifidum* positively influenced PCV13 antibody (PPS6B IgG) titers by mediating inflammation-related BTM activity. Collectively, these findings support the idea that early-life antibiotic exposure may induce a pro-inflammatory state, which hinders the colonization of beneficial microbiota like *Bifidobacterium*, and compromises optimal vaccine immunogenicity.

Ng *at al*. found a higher abundance of *B. adolescentis* in high responders.^28^ This bifidobacterial enrichment was linked to an increase in the pathways involved in carbohydrate metabolism. Similarly, Moroishi *et al*. reported an association between TT vaccine response and the CDP-diacylglycerol biosynthesis I and II pathways, with CDP-diacylglycerol being a key component of lipid metabolism.^42^ Ng *et al*. also observed a positive correlation between the enrichment genes involved in flagellin and pili production, and IgG levels post BNT162b2 and Sinovac vaccination. This relationship between cell motility genes and antibody response was corroborated by Daddi *et al.*, who found gene markers for flagellin, pili and capsular polysaccharides were positively correlated with final levels of IgG post BNT162b2 and Spikevax vaccination.^34^ These findings collectively highlight the potential role of carbohydrate and lipid metabolism, as well as cell motility and polysaccharide biosynthesis, in modulating immune responses to vaccines.

### 2.5. Limitations

The majority of human studies thus far have been observational, which precludes establishing causality between microbiota, metabolomic changes and vaccine immune responses. Moreover, some studies had small sample sizes, and the ethnic and geographic disposition of participants may have limited their ability to identify generalizable correlates of adaptive immune responses. In many studies, microbiota analysis was conducted via 16S rRNA gene amplicon sequencing rather than by shotgun metagenomics, allowing for examination of taxonomic profile differences in the gut microbiota between high and low antibody responders at the genus level, but not at the species or strain level. Similarly, the distinction between IgG antibody titers and neutralizing antibody assays reflects different aspects of the immune response. IgG levels indicate exposure and general antibody production, whereas neutralizing antibodies specifically measure functional immunity against viral infections. These methodological differences limit cross-study comparability and may explain inconsistencies in reported microbiota-immune associations. Moreover, most analyses of bacterial taxa rely on relative abundances, which are influenced by the abundance of other taxa and the further reliance on 16S rRNA amplicon sequencing to measure taxonomic abundance, with limited integration of functional data such as metagenomics, metabolomics or transcriptomics, which remains a key limitation. Due to the high degree of functional redundancy within the gut microbiota, changes in taxa do not necessarily demonstrate how immune responses to vaccinations will be impacted. Similarly, without measuring fecal SCFA levels, immune modulatory effects cannot be reliably inferred from taxonomic relative abundances alone, given SCFA production capacity varies significantly even among closely related bacterial species. Future studies using absolute quantification are needed and functional profiling will likely provide further mechanistic insight into the influence of the gut microbiota on vaccine immunogenicity. Function-focused approaches should be prioritized to better understand biological mechanisms and identify microbial targets for novel therapeutics. Additionally, diet plays a key role in influencing both microbiota composition and metabolite production. While most studies have not adjusted for dietary habits, recent papers by Lunken *et al*. and Ng *et al*. have begun exploring and accounting for diet^36,39^ - an important consideration for future work given the central role of diet in modulating microbiota composition and function, as well as direct dietary effects on immune responses.

## 3. Experimental model data (in vitro/animal studies)

### 3.1. Overview

**Figure 2.**
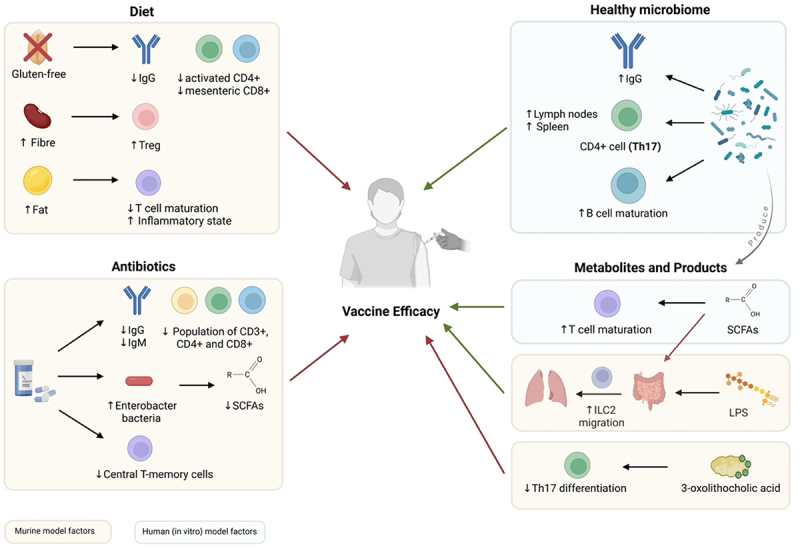
Microbiota mediated factors influencing vaccine efficacy. The diagram highlights how the healthy microbiome, its metabolites, dietary changes and antibiotic-induced dysbiosis can modulate the immune cell composition and therefore impact vaccine efficacy. Green arrows represent a positive impact (i.e. an increase); red arrows represent a negative impact (i.e. a decrease). Created in BioRender. Roberts, L. (2025) https://BioRender.com/rs9azln

A growing body of research is being conducted on the relationship between gut microbiota and its effects on both the adaptive and innate immune systems in the context of vaccination. Most studies focus on antibiotic-induced changes in the gut microbiota composition using murine models. Other research has explored the effects of dietary modifications, microbiota-derived metabolites, and germ-free (GF) conditions on immune cell composition and function. The findings from these studies shed light on how gut microbiota influences T and B cell responses, which are essential for vaccine-induced immunity (Figure 2). Understanding the relationship between gut microbiota composition and the immune system at cellular and molecular levels provides foundational knowledge for optimizing vaccine efficacy.

### 3.2. Germ-free models – microbiota-free animals

Germ-free (GF) mice have lower numbers of CD4^+^ cells and defective mesenteric lymph nodes and spleens.^65^ They have a reduced Th1 immune response with a clear bias toward Th2 responses. Their spleens are smaller and have fewer germinal centers, which are common sites for B-cell maturation, resulting in severely reduced natural IgG serum levels. The impact of the low natural antibody levels was seen when GF mice were injected with the PCV13 vaccine, which resulted in significantly lower PCV13-specific IgG and IgM responses.^43^ GF mice are also lacking in intestinal Th17 response,^66^ and such mice colonized with intestinal bacteria display increased Th17 cell numbers.

### 3.3. Microbiota-derived metabolites and products

The bacteria that make up the gut microbiota produce various metabolites with immunomodulatory properties. The changes in the immune cell composition can then impact the body’s response to the vaccines, as discussed above. For example, some bile acid secondary metabolites, such as 3-oxolithocholic acid, have been found to bind to RORγt, the key transcription factor of Th17 cells, inhibiting their differentiation,^66^ whilst SCFAs promote naïve T-cell differentiation to Th1 and Th17 cells and increase IL-10 expression.^67^ IL-10 promotes Treg formation and forms a negative feedback mechanism for SCFA-promoted T-cell generation and differentiation.^68^ Hence, SCFAs produced by the gut microbiota promote effector and Treg cell differentiation, thus having the potential to both stimulate and regulate the immune response to vaccinations.

However, a paper from 2021 reported some contradictory data comparing metabolite effects on innate and adaptive immune functions.^69^ Increased SCFA levels, such as butyrate, were associated with decreased intestinal innate lymphoid cell (ILC) proliferation and migration to the lung in influenza A infection.^70^ ILC2 cells play a role in modulating respiratory viral infection by differentiating into ILC1 cells, which subsequently enhance antiviral responses and have been shown to improve response to influenza vaccines.^71,72^ Mice with reduced ILC2 function and defective migration of dendritic cells to the lungs due to antibiotic-driven dysbiosis were injected with lipopolysaccharides (LPS), produced and found in Gram-negative bacteria, which corrected the immune function in influenza A infection and may also improve response to vaccination.^73^ The gut microbiota metabolites, such as SCFA, can have both pro-inflammatory and anti-inflammatory effects during acute infection and alter immune cell composition, impacting response to vaccinations. The ILCs have SCFA receptors at different concentrations depending on their origin, hence it is challenging to determine the overall impact SCFA has on the immune cell composition and on vaccine response.^69^ The reduction of ILC proliferation and migration due to increased SCFA levels was also mainly observed in patients with COPD, having a beneficial effect on their disease activity.^70^ It is therefore also crucial to investigate if this effect is seen in healthy individuals across various age groups. Although animal models show promising results in improving immune function with certain bacterial products or metabolite supplementation, further research is warranted to investigate the ideal concentrations for therapeutic effects, such as optimum vaccine efficacy.

### 3.4. Dietary changes

Gut microbiota composition is closely linked to dietary intake, with bacterial taxa and their metabolite concentrations heavily influenced by interventional diets, such as high fiber, high protein or high fat.^74^ Hence, dietary changes and their effects on vaccine efficacy have been investigated using murine models. In one study, mice on a gluten-free diet received the TT vaccine, and their IgG titers and immune cell levels were investigated.^75^ These mice had significantly lower antibody levels than the control group. They also had reduced levels of T-cells, specifically CD8^+^, in their mesenteric lymph nodes and reduced numbers of activated CD4^+^ cells. However, in their spleen, both CD4^+^ and CD8^+^ cells were more abundant.

Another study found that high-fiber diets were associated with higher levels of Treg cells, whilst high-fat diets were associated with low-grade inflammation and increased numbers of intraepithelial lymphocytes.^76^ Mice with diet-induced obesity were vaccinated against influenza and injected with the H1N1 virus, which showed different immune responses to their control group.^77^ The number of B cells produced was similar. However, the obese mice had more severe systemic inflammation, as T-cell maturation to memory cells was limited. The prolonged maturation of T-cells into memory cells caused prolonged activation of the T-cells and, hence, an extended inflammatory state in the mouse. The metabolic dysfunction of the T-cells was reversible if mice switched their diet back to the control diet prior to vaccination, leading to a 100% survival rate compared to 0% in continuously obese mice. Similarly, mice with diet-induced obesity and diabetes mellitus vaccinated against COVID-19 were found to have increased neutrophil levels in the lungs post-infection.^78^ Although antibody titers to the vaccine were similar to the control group, these mice had reduced viral clearance and higher mortality rates, suggesting decreased vaccine efficacy. These studies demonstrate that dietary alterations may impact the gut microbiota significantly to cause variations in the immune response and hence impact vaccine-induced immunity.

### 3.5. Antibiotic-induced microbiota perturbation

Various studies investigated how antibiotic-induced perturbations of the gut microbiota affect parenteral vaccine efficacy. It should be noted that broad-spectrum antibiotics may directly impact immune cell function, including that of lymphocytes and macrophages, independent of microbiota changes,^79,80^ but here we will focus on research linking antibiotic-driven microbiota perturbation to impaired immune response to vaccination. Kihl *et al*. investigated IgG titers and immune cell levels in murine models that were fed ampicillin and received the TT vaccine and booster.^75^ The antibiotic-treated mice had lower numbers of Bacteroidota and significantly lower antibody levels than untreated mice. They also had impaired peripheral immunity, demonstrated by their reduced fractions of CD3^+^ and CD4^+^ cells in the mesenteric lymph nodes and lower numbers of activated T-cells in the spleen. The results indicate that antibiotic-induced perturbation of the gut microbiota can lead to lower antibody levels and reduced overall efficacy of the TT vaccine.

Similarly, a study by Oh *et al*. from 2014 showed that mice treated with antibiotics or raised germ-free had significantly reduced IgG responses to the inactivated influenza vaccine.^81^ This was due to low levels of Toll-like receptor five (TLR5). When the researchers restored microbiota using flagellated commensals or directly administered purified flagellin, vaccine responses improved. This finding demonstrates that microbiota-derived flagellin signals via TLR5 are essential for effective humoral immunity.

Interestingly, another study investigating vaccine efficacy in mice showed that both antibiotic-treated and GF mice did not significantly decrease their IgG responses to the BNT162b2 vaccination.^82^ The difference in T-cell response was also not significant, and neither was the number of B-cells or Th-cells in the vaccine-draining iliac lymph nodes.

In a study where young mice received the BCG vaccine, their IgG1, IgG2c and IgM antibody levels were lower if they had been exposed to ampicillin and neomycin during gestation.^83^ When giving a booster dose, the antibody levels did not improve. Their antibody levels were also reduced to other vaccinations commonly given to infants during their routine vaccination schedule, such as Meningitis B and C, PCV13 and the Hexavalent combination vaccine. The mice had increased levels of *Enterobacter*, which can be a common microbiota change following antibiotic exposure.^84^ An overgrowth of *Enterobacter* bacteria has been associated with the suppression of SCFAs and an increase in LPS production.^85^ Interestingly, adult mice who received the same antibiotic dosages as the mothers during pregnancy had a normal immune response to the PCV13 vaccine, even though the antibiotic-driven microbiota perturbation was similar. There were also no changes in the IgG levels of adult mice continuously exposed to antibiotics compared to the control group. Young antibiotic-treated mice that received FMT from the control group did not have impaired IgG responses to the PCV13 vaccination. These results show the vulnerability of the gut microbiota at early stages of life, especially during gestation. It also provides further insight into the possibility of microbially targeted therapeutics as management for antibiotic-induced microbiota perturbation and improvement in vaccine responses.

In addition to altering metabolite concentrations, an overgrowth of *Enterobacter* leads to an imbalance of the T-cell population, specifically Th1, Th2, Th17 and Treg cells.^86^ Treg cells develop in recipients of vaccinations to regulate the immune response when they get infected, commonly seen in vaccinated COVID-19 patients.^87^ This imbalance in T-cells may lead to the development of autoimmune and inflammatory diseases, as well as reduce vaccine-induced immunity and therefore lead to higher infection rates and more severe infections.

A recent study by Feng *et al*. demonstrated that both antibiotic-treated and germ-free mice receiving the rabies vaccine had reduced antibody production and T-helper cell populations in draining lymph nodes.^88^ The mice also showed a Th1-biased immune response, disrupting the balance that is required for optimal germinal center and memory B cell formation. Additionally, antibiotic-induced perturbations of the microbiota resulted in lower concentrations of secondary bile acids, like deoxycholic acid. However, supplementing these metabolites restored vaccine immunogenicity. The study also showed that broad-spectrum antibiotic use in humans significantly impairs both primary and memory immune responses to the rabies vaccine in healthy adults. The participants had impaired B-cell activation and reduced antibody titers, which lasted up to 180 days post vaccination. These findings provide mechanistic evidence that bile acid signaling may be a key link between vaccine immunogenicity and the gut microbiome.

Lastly, a study investigated the impacts that perturbation of the microbiota had on lung immune cell infiltration. Mice were vaccinated with the BCG or L91 tuberculosis vaccine and given a booster after 15 days.^89^ Sixty days after inoculation, one group of mice received an antibiotic cocktail orally consisting of amphotericin B, trimethoprim, polymyxin, vancomycin and carbenicillin, followed by exposure to *Mycobacterium tuberculosis* via the air 21 days later. The antibiotics were continued to maintain microbiome dysbiosis. The antibiotics-treated mice had a significantly reduced number of taxa, showing a reduction in microbiota diversity. There was a decrease in the number of *Bifidobacterium* and *Lactobacillus* families, but an increase in *Bacteroides*. Both CD4^+^ and CD8^+^ cells were less proliferated in the antibiotic-treated mice. The intervention group had lung-specific immune differences compared to the control group. They had a significantly higher disease burden in their lungs. There was a lower activation of T-cells in the lung with reduced expression of the CD44 and CD127 activation markers and reduced effector CD4^+^ and CD8^+^ memory T-cell populations. There was also a significant reduction in resident memory T-cells, which play an important role in the protection from respiratory pathogens such as tuberculosis or respiratory syncytial virus.^90^ The antibiotic-treated mice had drastically reduced central T-memory cells in the spleen and lymph nodes, which are the homing sites. This work demonstrates that antibiotic-induced dysbiosis delays vaccine-induced immune response and impacts the immune function at the primary site of infection and secondary lymphoid organs by impairing systemic T-cell function.^79,80^

## 4. Future directions

In this review, we have discussed evidence linking the composition and function of the gut microbiota to immune responses to vaccination, raising the question whether modulating the microbiota (or its products) might be a means to improve vaccine efficacy. A number of experimental approaches have been assessed, including dietary modification, co-stimulation with pro-inflammatory LPS and even FMT.^83,86,91^ A recent study showed promising results in GF mice, where *B. bifidum* and *Lactobacillus acidophilus* were administered a week before PCV13 vaccination and resulted in significantly increased vaccine-specific antibody levels.^43^ In human studies, both probiotics (cocktails of bacteria with supposedly beneficial properties) and prebiotics (non-digestible products that promote probiotic microbiota growth) have been investigated for their potential to improve vaccine response.^92^ Clinically, commonly used probiotics include *Bifidobacterium*, *Lactobacillus*, non-lactic acid bacteria and yeasts.^91^ Common prebiotics, including inulin, fructo-oligosaccharides (FOS), and galacto-oligosaccharides (GOS), have been linked to increasing *Bifidobacterium and Lactobacillus* numbers and gut IgA titers in animal models.^93–95^ *Bifidobacterium infantis* and *Lactobacillus* show evidence of moderating immune function and reducing secondary infection in COVID-19.^31,96^

Similarly, a six week course of *Bifidobacterium lactis* and *Lactobacillus paracasei* following the influenza vaccine resulted in higher IgG and IgA titers and seroconversion rates.^97^ Another study found the use of *L. paracasei, Lactobacillus bulgaricus*, and *Streptococcus thermophilus* over 13 weeks also led to higher IgG levels and seroconversion rates several weeks post influenza vaccination.^98^ Indeed, meta-analysis indicates that probiotics may have a role in boosting responses in vulnerable older people, but larger, high-quality trials are needed.^99^ Evidence suggests the use of concomitant pre- and probiotics can enhance hemagglutination inhibition antibody titers post influenza vaccination.^100^ There are rare cases of bacteremia when using probiotics in immunocompromised individuals, but as Yeh *et al.’s* subgroup analyses showed similar effects between prebiotic and probiotic supplementation, prebiotics could be considered an alternative.^101^

Other probiotic preparations including *Lacticaseibacillus rhamnosus GG (LGG)*, *Lactiplantibacillus plantarum* and *Pediococcus acidilactici KABP021* have shown promise in reducing the severity of active COVID-19 infection. Participants in multiple placebo-controlled trials experienced delayed onset of COVID-19 and fewer symptoms, including post-COVID syndrome.^102–104^ However, Jesperson *et al*. found *L. paracasei* only had positive symptomatic effects.^105^ LGG has also had positive results in influenza vaccinees, with increased IgA and IgG titers following the use of *LGG, L. plantarum and Lactobacillus fermentum*.^106,107^ Moreover, maternal *LGG* supplementation showed a decrease in specific antibody responses in TT, *Haemophilus influenzae* type b (Hib), and PCV vaccines in infants, showing there is a scope for modulation, but it was not beneficial in improving vaccine-specific immunity.^108^ Collectively, probiotics demonstrate significant potential in regulating gut microbiota and thereby enhancing immune function post vaccination, although a full exploration of this potential is beyond the scope of this review.

In addition to probiotics, synbiotics, which combine probiotics with prebiotics to allow optimal growth of beneficial microbial cultures, have been investigated for modulating the gut microbiota and thus enhancing vaccine immunogenicity. A double-blinded, human randomized controlled trial found that supplementation of *Bifidobacterium longum* with gluco-oligosaccharide had no significant effect on B-cell or T-cell responses to seasonal influenza vaccinations.^109^ To date, only a limited number of human trials have evaluated synbiotics in this context, with no evidence of a significant benefit of synbiotic supplementation on vaccine response. Further research is required to better understand the mechanisms by which synbiotics may influence vaccine immunogenicity and to identify optimal strain concentrations. Similarly, although animal models have shown promising results in using FMT trials to improve vaccine immunogenicity,^24,110^ these findings have yet to be validated in humans. Additionally, microbiota-based interventions remain unexplored across certain vaccine types, including the mRNA vaccine platforms, which have unique mechanisms of action and therefore may require tailored strategies.

Finally, despite growing interest in these approaches, the implementation of microbiota screening or manipulation into routine vaccine programs presents logistical and ethical challenges. Implementing large-scale microbiota screening would require a significant investment in infrastructure and bioinformatics capacity. Microbiota modulation methods for example, with FMT, are currently costly and not routinely available outside of specialist centers, making them inaccessible in the context of population-wide vaccination programs administered via primary care.^111^ Moreover, with vaccine hesitancy and non-uptake being the key barriers to effective herd immunity against common pathogens, the introduction of additional hurdles aimed at improving vaccine efficacy might actually result in poorer levels of immunity overall.^112^

## 5. Conclusion

In this review, we have examined the evidence linking the gut microbiome to immune responses to parenterally delivered vaccines. We have explored research in pre-clinical models, which indicates that dysbiosis of the gut microbiota can negatively impact such responses. These show the gut microbiota playing a role in modulating T-cell subsets, antibody production, and immune regulation. Moreover, there is now a body of evidence from human studies associating the composition and function of the gut microbiota with functional readouts of vaccine-induced immunity. For example, taxa such as *Bifidobacterium* are consistently associated with enhanced antibody responses across different vaccine types. These data are largely from observational studies, and further longitudinal and interventional studies are needed, for example, deploying dietary interventions or microbially targeted therapeutics alongside vaccination in the appropriate setting. Dysbiosis induced by antibiotics or dietary changes may disrupt immune responses and alter the immune cell composition, especially in early life. Thus, greater awareness is also needed of the potential negative impact of antibiotics on vaccine responses, emphasizing the importance of antibiotic stewardship around the time of immunizations, particularly in children.

## Data Availability

Data sharing is not applicable to this article as no new data were created or analyzed in this study.
